# An open letter to the Bureau Approving Officials of the United States government

**DOI:** 10.1016/j.ohx.2026.e00825

**Published:** 2026-08-19

**Authors:** Joshua M. Pearce

**Affiliations:** Western University, 601 M&M Building, Houghton MI 49931, United States

**Figure:**
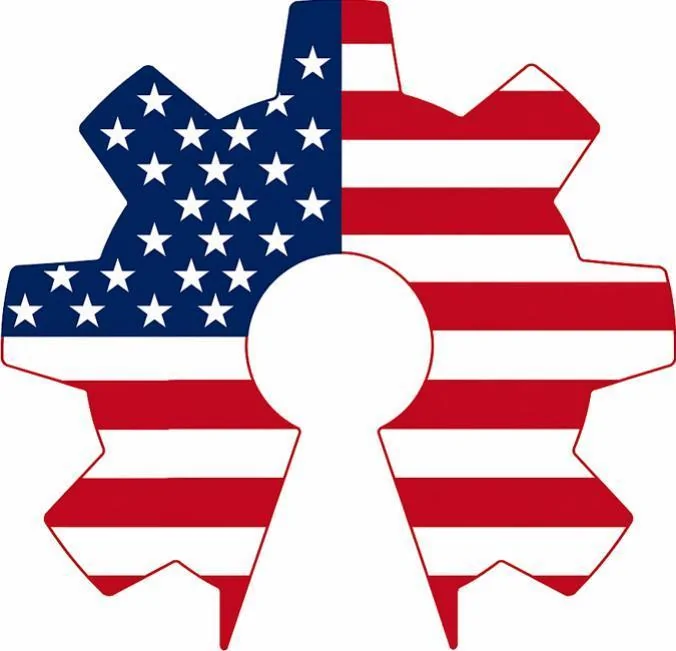

From: The Editor-in-Chief, *HardwareX*  

To: Bureau Approving Officials (BAOs), United States Government Agencies

Subject: The Scientific and Economic Imperative of Open Source Hardware and the Danger of Bureaucratic Censorship.

To the Bureau Approving Officials of the United States Government:

I write to you today in my capacity as the Editor-in-Chief of HardwareX [1], the top peer-reviewed scientific journal dedicated to the open-source design of scientific equipment. It has recently come to my attention that a deeply concerning trend is emerging within the ranks of Bureau Approving Officials (BAOs) across various U.S. federal agencies. Specifically, BAOs are requesting that federal researchers redact or entirely remove critical information from their manuscripts prior to publication. These requested redactions target comparative cost analyses of the bill of materials (BOMs) and language that officials fear could be construed as “endorsement, disparagement, or competition with the private sector.” A peer-reviewed comparison of cost, reproducibility, and performance is not an endorsement any more than a methods comparison paper is an endorsement; it is part of the scientific record needed for replication, evaluation, and efficient public spending.

To be unequivocal: demanding the removal of product cost data, BOMs and economic comparisons from scientific literature is methodologically invalid, economically detrimental (both anti-competition/anti-free market) and represents a severe disservice to the American taxpayer.

## 1 The paradigm of open hardware

To understand why these censorship requests are so damaging, one must first understand the nature of open hardware. *HardwareX* publishes only open hardware designs. Open-source hardware is not a rejection of commerce; by definition it permits others to study, modify, distribute, make, and sell hardware based on the design [2]. This means that the hardware detailed in our journal is licensed in such a way that anyone (*including private enterprises*) can freely use, study, modify, distribute, and *commercialize* the designs [2].

The open hardware ecosystem is heavily supported by industry itself. Globally the best example is actually American: The Open Source Hardware Association (OSHWA) is an United States-based organization comprised primarily of small and medium-sized enterprises (SMEs) that leverage open-source business models for commercial success [3], [4], [5], [6]. By publishing in *HardwareX*, federal researchers are not competing with the private sector; they are directly supporting it. They provide free, rigorously tested, and peer-reviewed research and development to American SMEs, enabling these businesses to commercialize new products without the crippling overhead of initial prototyping and design [7]. There is a massive, well-documented precedence for the commercialization of open hardware driving innovation and job creation and is so beneficial some authors have called for all government funded research to be open source by default [8].

## 2 Stretching tax dollars and enabling domestic manufacturing

The economic argument for open hardware in science is unassailable. Peer-reviewed research has repeatedly demonstrated that open-source scientific hardware simply costs less [9], [10]. A recent *HardwareX* review found average savings of 87% overall, rising to 92% for 3-D-printed tools and 94% for tools combining 3-D printing with Arduino-class electronics, while earlier case studies often reported savings in the 90–99% range [11].

Currently, substantial U.S. tax dollars are expended annually to purchase costly, proprietary scientific tools, often manufactured internationally. When federal researchers develop and publish an open-source alternative, they allow U.S. tax dollars to stretch exponentially further. When Finland looked at moving to open hardware, they found Finland’s science funders could save between 2.84 and 27.7 million € per year with open source purchasing [12]. This is small when compared to America, which conservatively spends billions per year on scientific equipment. Open scientific equipment provides extra leverage as it enables lower-cost and faster innovation across any scientific area that it includes. Again, conservatively U.S. scientific R&D could go 10× faster with 10x more equipment at the same cost if moved to open hardware. A federal grant that could previously only afford a single proprietary instrument can now fund an entire laboratory of open-source equivalents, vastly accelerating the pace of discovery [13]. These kinds of efficiencies are exactly what fiscally conservative governments should focus on for maximum impact of tax investments.

Furthermore, open hardware enables U.S. domestic manufacturing in the most extreme and resilient sense. By utilizing distributed manufacturing technologies such as 3D printing, CNC milling, laser cutters, and open-source microcontrollers, open hardware allows scientific equipment to be manufactured locally, on-demand, within the United States [9]. This distributed approach insulates scientific supply chains from global disruptions and repatriates manufacturing capabilities [14].

## 3 Censoring is Anti-Science and Inconsistent with U.S. Policy

This brings me to the core issue: the request by BAOs to remove cost reporting and language perceived as “disparaging” to the private sector.

Science is fundamentally about the pursuit of truth, and in the realm of engineering and hardware design, cost is a critical, quantifiable variable [15]. To mandate the removal of cost data is to force researchers to publish incomplete research. Requests to remove empirically grounded cost and design information are difficult to reconcile with the U.S. government’s broader public-access and open-science direction, which now emphasizes free, immediate access to federally funded research outputs and the data underlying them [16], [17].

For example, stating that an open-source syringe pump can be built for $50 in any American lab while an internationally-manufactured proprietary equivalent costs $1,500 (or even up to $2500) is not “disparagement” – it is an empirical fact [18]. Hiding this fact to protect the profit margins of legacy corporations is an act of intellectual timidity and frankly un-American. To illustrate the magnitude of this impact: that specific open-source syringe pump design library example was downloaded thousands of times within its first months of publication, resulting in a calculated downloaded substitution valuation of between about $1–12 million in savings to the scientific community in just a single year [19]. Since then, those designs (developed in America) have been downloaded tens of thousands of times, conservatively savings scientists 10 s of millions of dollars. Censoring such data prioritizes the feelings of monopolistic vendors over the efficiency of the U.S. government researchers, science as a whole and the success of American SMEs. Federal researchers should report on how they are saving taxpayer money, and BAOs should be championing these savings. Instead of censoring it, consider press releases and awards for your top scientists that make open hardware breakthroughs that scale across all of science.

## 4 Precedent: American open hardware companies and commercial success

The fears of a few BAOs are entirely unfounded when viewed against the historical record. There is a rich precedent of government officials and researchers publishing open hardware designs without causing the collapse of the private sector; rather, it has resulted in highly cited, impactful science that directly benefits the public [20] and seeded an entire generation of successful American businesses. Open hardware is NOT anti-business – it is very much pro-business.

The American open hardware business ecosystem is robust and thriving. Examples of successful U.S.-based companies built on open-source hardware principles that interact with *HardwareX* include:

- Arduino USA is owned by the large technology firm Qualcomm (revenue of $44B/year and headquartered in San Diego, California). Arduino’s open-source microcontroller platform has become the de facto standard for embedded prototyping in laboratories worldwide and underpins countless *HardwareX* publications.
- SparkFun Electronics (Boulder, Colorado), a U.S. SME that has grown to a US$32 million in annual revenue business with 146 employees by selling open-source electronic components and educational kits [21], which again have been used in dozens of *HardwareX* articles.
- Similarly, *HardwareX* authors often use components from Adafruit Industries (New York City), which generates over $45 million in annual revenue selling open-source hardware while employing dozens of American workers.
- Other companies like Backyard Brains (Ann Arbor, Michigan), which commercialized open-source neuroscience tools was originally described in peer-reviewed open hardware literature [22] and used by *HardwareX* authors.
- OpenTrons (Long Island City, New York), which democratized laboratory automation through open-source liquid-handling robots, directly displaced proprietary systems costing an order of magnitude more. *HardwareX* authors use it as a platform to extend functionality in their labs [23].
- LulzBot is headquartered and manufactured in Fargo, North Dakota, is an entirely open-source 3D printer manufacturer that demonstrated that open hardware can compete directly with proprietary incumbents in domestic manufacturing is actually used by the U.S. Marines to “Improvise, Adapt, and Overcome” [24] in addition to the labs of dozens of *HardwareX* authors that need custom components as well as their scientific line of bioprinters designed specifically for U.S. scientists. Sometimes these companies are only scientific equipment adjacent. For example, re:3D (Austin, Texas) is a 3D printing spin off from NASA engineers and MatterHackers (Lake Forest, California), also built thriving U.S. businesses around the open-source RepRap 3-D printing ecosystem [25], [26], [27].

These companies and other open hardware firms collectively employ thousands of Americans and generate hundreds of millions of dollars in annual revenue—all built upon the foundation of openly licensed designs [28], [29]. When a federal researcher publishes a low-cost environmental sensor or a novel diagnostic tool in *HardwareX*, they are planting the seeds for the next generation of American hardware startups.

## 5 Conclusion

The policy of censoring cost data and economic comparisons in federal research is anti-science, anti-taxpayer, and anti-innovation. It stifles the very mechanisms that allow open hardware to drive domestic manufacturing and empower American SMEs.

I strongly urge all Bureau Approving Officials to

• permit empirically grounded BOMs and cost comparisons in manuscripts;
• issue written guidance distinguishing comparative scientific analysis from endorsement;
• align manuscript review with agency public-access plans and broader Office of Science and Technology Policy open-science expectations.

Consider celebrating the open hardware paradigm. Allow your researchers to proudly report how they are innovating, saving taxpayer funds, and providing invaluable open-source R&D to the American public.  

Sincerely,  

Joshua M. Pearce, PhD

The Editor-in-Chief

*HardwareX*

Elsevier

## Declaration of competing interest

The author is the Editor-in-Chief of *HardwareX* and was not involved in the editorial handling or publication decision for this manuscript.
